# Detection of ESBL-producing *Klebsiella oxytoca* complex with VITEK 2 system and screening cutoffs for implementing confirmatory tests

**DOI:** 10.1128/jcm.00128-25

**Published:** 2025-05-09

**Authors:** Edgar I. Campos-Madueno, Gisele Peirano, Claudia Aldeia, Maria V. Elzi, Claudine Kocher, Laurent Poirel, Patrice Nordmann, Vincent Perreten, Johann D. D. Pitout, Andrea Endimiani

**Affiliations:** 1 Institute for Infectious Diseases (IFIK), University of Bern 87618, Bern, Canton of Bern, Switzerland; 2 Division of Microbiology, Alberta Precision Laboratories 590576 , Calgary, Alberta, Canada; 3 Pathology and Laboratory Medicine, University of Calgary 574842, Calgary, Alberta, Canada; 4 Medical and Molecular Microbiology, Department of Medicine, University of Fribourg 30484, Fribourg, Switzerland; 5 National Reference Center for Emerging Antibiotic Resistance (NARA), Fribourg, Switzerland; 6 Institute of Veterinary Bacteriology, University of Bern 30398, Bern, Switzerland; Cleveland Clinic, Cleveland, Ohio, USA

**Keywords:** *Klebsiella oxytoca*, VITEK 2, detection, ESBL, OXY, CTX-M, AST, cutoffs

## Abstract

*Klebsiella oxytoca* complex (*Ko*C) are important nosocomial pathogens that can be reservoirs of transmissible extended-spectrum β-lactamase (ESBL) genes. Therefore, it is essential for clinical microbiology laboratories to distinguish between *Ko*C producing ESBLs (ESBL-*Ko*C) and those hyperproducing the natural OXY-type β-lactamases (hOXY-*Ko*C). We investigated the abilities of VITEK 2 with and without using the Advanced Expert System (AES) to detect ESBL producers among 44 well-characterized *Ko*C strains (including 11 ESBL-*Ko*C and 21 hOXY-*Ko*C). VITEK 2/AES showed 100% sensitivity (Se) and 64.7% specificity (Sp), whereas the VITEK 2 coupled by the Clinical Laboratory Standards Institute (CLSI) ESBL confirmatory tests (ESBL-CTs; i.e., disk-combination tests) showed 100% Se and 97.5% Sp to detect ESBL-*Ko*C. We also analyzed *Ko*C-specific screening cutoffs for ceftriaxone (CRO), cefpodoxime (CPD), ceftazidime (CAZ), cefotaxime (CTX), and aztreonam (ATM) to negate unnecessary ESBL-CTs. As a result, we propose the following screening cutoffs (minimum inhibitory concentration [MIC] and inhibition zone diameter): CRO, >4 µg/mL and ≤16 mm; CPD, >4 µg/mL and ≤10 mm; CAZ, >1 µg/mL and ≤22 mm (European Committee on Antimicrobial Susceptibility Testing [EUCAST] disk)/≤30 mm (CLSI disk); CTX, >4 µg/mL and ≤12 mm (EUCAST disk)/≤22 mm (CLSI disk); ATM, >1 µg/mL and ≤28 mm. Notably, all suggested cutoffs could assure 100% Se and high Sp/positive predictive values for our 44 *Ko*C strains. In conclusion, the AES performed poorly, while VITEK 2 with the CLSI ESBL-CTs yielded a reliable methodology to distinguish ESBL-*Ko*C from hOXY-*Ko*C. This study also proposed revised screening cutoffs for detecting ESBL-*Ko*C and reducing the unnecessary use of ESBL-CTs.

**IMPORTANCE:**

Species within the *Klebsiella oxytoca* complex (*Ko*C) are emerging clinical pathogens of increasing concern. These bacteria can acquire plasmid-mediated ESBL genes, seriously complicating antibiotic treatment and overall management of infected patients. Differentiating ESBL-producing from non-ESBL-producing *Ko*C isolates is therefore crucial. However, this task presents significant challenges for clinical laboratories. In this work, we showed that the automated VITEK 2 system equipped with its AES fails to differentiate the two groups of *Ko*C isolates. In contrast, VITEK 2 alone followed by the ESBL screen and phenotypic confirmatory tests provides accurate differentiation. Since this latter approach increases the diagnostic workload, we also proposed new screening cutoffs for key cephalosporins that may reduce the current high number of unnecessary confirmatory tests.

## INTRODUCTION

Bacteria belonging to the *Klebsiella oxytoca* complex (*Ko*C) are responsible for numerous types of infections, including those being hospital-acquired (1, 2). These pathogens produce the chromosomally encoded and non-transmissible class A OXY-type β-lactamases (formerly named K1) that are expressed at different levels according to the genetic asset of their promoter region (3, 4). Produced at low level, wild-type (WT) OXYs confer resistance to penicillins, whereas their overexpression guarantees hydrolysis of extended-spectrum β-lactams: efficient for ceftriaxone (CRO) and aztreonam (ATM), partial for cefotaxime (CTX), and weak for ceftazidime (CAZ) (1). However, OXY variants capable of hydrolyzing CAZ at high levels (e.g., OXY-2–5, OXY-2–15) have also been reported (1, 5, 6).

*Ko*C isolates may also acquire multidrug resistance plasmids carrying clinically relevant antimicrobial resistance genes (ARGs) such as the extended-spectrum β-lactamases (ESBLs; mainly of CTX-M type) that significantly limit our therapeutic options to a few antibiotics (e.g., to carbapenems) (3, 7–15). Therefore, identification of ESBL-producing *Ko*C is of clinical and public health importance, since these strains are difficult to treat, have the potential to cause hospital outbreaks, and possess the ability to exchange ESBL genes via conjugation of their plasmids with other bacterial species (1, 2, 7, 16, 17).

Recently, we have shown that standard antimicrobial susceptibility testing (AST) is unable to discriminate between *Ko*C strains producing ESBLs (ESBL-*Ko*C) and hyperproducing OXYs (hOXY-*Ko*C) (3). ESBL and OXY β-lactamases are inhibited by clavulanate (CL) (1); therefore, minimum inhibitory concentration (MIC)-based ESBL confirmatory tests (ESBL-CTs) failed to distinguish between ESBL and those hyperproducing OXY *Ko*C strains (i.e., high false-positive rates). On the other hand, the combined-disk tests (CDTs) and the double-disk synergy test (DDST) performed according to the European Committee on Antimicrobial Susceptibility Testing (EUCAST) guidelines demonstrated high accuracy (3).

In this context, the EUCAST and the Clinical Laboratory Standards Institute (CLSI) recommend ESBL screening cutoffs for CRO, CTX, CAZ, and cefpodoxime (CPD) that are identical for *Escherichia coli*, *Klebsiella* spp., and other Enterobacterales; the CLSI also indicates the use of ATM (18, 19). However, these approaches could not offer the best screening performance for specific species such as those belonging to the *Ko*C. For instance, by using the EUCAST criteria, Platteel et al. showed that the positive predictive value of the ESBL screening (i.e., CTX and/or CAZ MICs > 1 µg/mL) was 95% for *Klebsiella pneumoniae*, 76% for *E. coli*, but only 27% for *K. oxytoca* (*Ko*) (20).

Phenotypic detection of ESBL-*Ko*C strains can also be achieved using more rapid commercial automated AST systems (21). In particular, the VITEK 2 platform (bioMérieux) offers the option to use the Advanced Expert System (AES) software (bioMérieux) to interpret the results of AST cards and add comments about the possible mechanism(s) of antibiotic resistance. The analytic capacity of the AES is based on a large database that contains >3,500 phenotypes and >30,000 MIC distributions (21, 22). However, no specific study focusing on the ability of the VITEK 2/AES to detect ESBL-*Ko*C strains has been performed so far. Until now, information on this matter can be retrieved only from several older studies that tested mixed collections of Enterobacterales in which the number of *Ko* strains was small and their molecular characterization rather limited (23–27).

Here, a well-characterized collection of 44 *Ko*C strains was used to test the ability of the VITEK 2 with and without using the AES to detect a subgroup of isolates producing ESBLs. We also analyzed the CRO, CPD, CAZ, CTX, and ATM phenotypic results and proposed specific *Ko*C screening cutoffs (MIC and disk inhibition zone diameters) that provided high sensitivity, but also reduced the number of unnecessary ESBL-CTs.

## MATERIALS AND METHODS

### Bacterial strains and characterization

Forty-four non-carbapenemase-producing *Ko*C isolates previously characterized at the Institute for Infectious Diseases (IFIK, Bern, Switzerland) were included in the present study (3). In brief, the 44 *Ko*C were unique isolates detected from humans and animals and routinely identified (ID) by using the matrix-assisted laser desorption ionization-time of flight mass spectrometry (Bruker). All strains underwent whole-genome sequencing (WGS) with the NovaSeq 6000 Illumina platform. High-quality *de novo* draft genome assemblies were generated with Unicycler v.0.4.8 and deposited in the NCBI GenBank public repository (BioProject: PRJNA894995) as previously described (3). Moreover, draft assemblies were used for species ID, ARGs, plasmid replicon type, and sequence type (ST) definition along with analyses of *bla*_OXYs_ and their promoter regions (full epidemiological and WGS data in File S1, page 1) (3). Strains also underwent ASTs implementing the gold-standard MIC ESB1F and GNX2F broth microdilution (BMD) Sensititre panels (Thermo Fisher Scientific). Strains were defined as hOXY-*Ko*C when the MIC of CRO was >1 µg/mL and lacked genes encoding for ESBLs (*bla*_ESBLs_) or plasmid-mediated AmpCs (*bla*_pAmpCs_). Sensititre ASTs were performed in duplicate, leading to consistent results (File S1, page 2) (3).

All *Ko*C with elevated CRO MIC (i.e., >1 µg/mL in BMD) were further analyzed with several CL-based ESBL-CTs (28). In particular, we implemented (i) CTX/CTX-CL and CAZ/CAZ-CL BMD assays included in the ESB1F Sensititre panel, (ii) MIC gradient strip tests (Liofilchem) with cefepime (FEP)/FEP-CL, (iii) the EUCAST-recommended ESBL CDT Kit (Liofilchem) with CTX/CTX-CL (5/5–10 µg), CAZ/CAZ-CL (10/10–10 µg), and FEP /FEP-CL (30/30–10 µg) disks, and (iv) the EUCAST-recommended DDST with CTX (5 µg), CAZ (10 µg), FEP (30 µg), and ATM (30 µg; Bio-Rad) disks placed with a distance center to center of 25 mm (DDST-25) and 30 mm (DDST-30) around a disk of amoxicillin-clavulanate (AMC; 20-10 µg; Bio-Rad). Mueller-Hinton (MH) broth (Thermo Fisher Scientific) or agar (Oxoid) was used, respectively (3). Results of BMD, CDT, and DDST assays were interpreted as ESBL-positive according to the EUCAST criteria (18). All ESBL-CTs were repeated two times, leading to consistent results (File S1, page 3) (3). ATCC strains *E. coli* 25922 and *K. quasipneumoniae* 700603 were used as quality controls.

### VITEK 2 system with the AES

The 44 *Ko*C strains were tested at the Laboratory for Clinical Microbiology of IFIK, which routinely uses the VITEK AST-N242 card on a VITEK 2 apparatus, with final reporting interpreted according to EUCAST criteria (28).

Fresh colonies (overnight) were picked from MacConkey II agar plates (BD BBL). As for routine use, the AES (version 9.04.4) of VITEK 2 was activated. The species ID obtained using the WGS output (i.e., *Ko*) was manually entered into the instrument in order to enable the AES to ascertain the phenotype. ATCC strains *E. coli* 25922 and *Pseudomonas aeruginosa* 27853 were used as quality controls.

*Ko*C strains were provided to the Laboratory for Clinical Microbiology of IFIK with coded names randomly assigned (e.g., *Ko*C #1, *Ko*C #2; see File S1). Only the principal investigator (i.e., A.E.) possessed the encoded list with the original strain names and did not participate in any processing step of the 44 *Ko*C strains to ensure independent and unbiased testing. The VITEK AST-N242 card assay was repeated if (i) the AES reported inconsistent AST results for the database phenotypes/MICs distributions (29) or (ii) the resultant CRO MICs were significantly different (i.e., ≥ or ≤ 3 twofold dilutions) compared to those obtained with the BMD assay using the Sensititre panels (i.e., after A.E. accessed the VITEK 2 results).

### VITEK 2 system without the AES

The same 44 coded *Ko*C strains were shipped by the IFIK to the Alberta Precision Laboratories (APL; Calgary, Canada) using swabs with transport medium (Transystem, Copan).

Fresh colonies (overnight) on blood agar plates (Dalynn Biologicals) were used to perform the ASTs implementing the VITEK AST-N390 card on a VITEK 2 apparatus and employing the CLSI criteria (19). The species ID (i.e., *Ko*) was manually entered into the instrument. ATCC strains *E. coli* 25922 and *K. quasipneumoniae* 700603 were used as quality controls.

At the APL, the AES of VITEK 2 is not routinely implemented. On the other hand, *Ko*C strains showing a VITEK 2 MIC >1 µg/mL for CRO and/or CAZ (i.e., CLSI ESBL screening criteria) are tested for ESBLs on MH agar plates (Oxoid) with the CLSI-recommended CTX/CTX-CL (30/30–10 µg) and CAZ/CAZ-CL (30/30–10 µg) CDTs (MAST Group). Results of each CDT are interpreted as ESBL-positive according to the CLSI criteria. One positive result is sufficient to define the *Ko*C strain as ESBL-positive (19). Strains that tested negative for ESBLs are reported as hOXY-*Ko*C.

### Disk diffusion assays

At the IFIK, disk diffusion analyses for CRO (30 µg EUCAST/CLSI disk; Bio-Rad), CPD (10 µg EUCAST/CLSI disk; Bio-Rad), CAZ (10 µg EUCAST disk; Liofilchem), CTX (5 µg EUCAST disk; Liofilchem), and ATM (30 µg EUCAST/CLSI disk; Bio-Rad) on MH agar plates (Oxoid) were also performed. Assays were repeated twice, leading to consistent results. On the other hand, at the APL, a single disk diffusion assay (MAST Group) for CAZ (30 µg CLSI disk), CTX (30 µg CLSI disk), and ATM (30 µg EUCAST/CLSI disk) was performed (19, 28).

### Statistical analyses

Sensitivity (Se) and specificity (Sp) for all ESBL-CTs and VITEK 2 results were calculated considering the WGS results as reference (File S1, page 1): true positives (the ESBL-*Ko*C strains by WGS analysis) and true negatives (the hOXY-*Ko*C and the WT-*Ko*C strains). Se, Sp, positive predictive values (PPV), and negative predictive values (NPV) were also calculated for the MICs and inhibition zone diameters (i.e., CRO, CPD, CAZ, CTX, and ATM) that could be used as alternative screening cutoffs to subsequently implement the ESBL-CTs.

## RESULTS AND DISCUSSION

### Features of *Ko*C strains

The previously obtained molecular and phenotypic characteristics of the 44 *Ko*C strains (11 CTX-M-1/-15 producers, 21 hOXY, and 12 WT), along with the performance of the ESBL-CTs, are summarized in Table 1 (full data in File S1) (3).

**TABLE 1:** Characteristics of the 44 *Ko*C strains. Assays performed at the Institute for Infectious Diseases (IFIK) ^*a*^

Strain/species^*b*^^,^^*c*^	Molecular characterization^*b*^		ESBL confirmatory tests (ESBL-CTs)^*b*^^,^^*e*^							VITEK AST-N242 card (MIC, µg/mL) with AES (9.04.4)^*g*^					CRO^*b*^		CPD^*b*^		CAZ^*b*^		CTX^*b*^		ATM^*m*^	
			EUCAST CDTs			EUCAST DDST^*f*^ (30 mm)	BMD		Strip						BMD^*d*^ (MIC, µg/mL)	30 µg disk (mm)	BMD^*d*^ (MIC, µg/mL)	10 µg disk (mm)	BMD^*d*^ (MIC, µg/mL)	10 µg disk (mm)	BMD^*d*^ (MIC, µg/mL)	5 µg disk (mm)	BMD^*d*^ (MIC, µg/mL)	30 µg disk (mm)
	β-lactamases (promoter of OXYs)	*Ko*C group^*d*^	CTX/ CTX-CL	CAZ/ CAZ-CL	FEP /FEP- CL		CTX/ CTX- CL	CAZ/ CAZ- CL	FEP/ FEP- CL	PTZ	CPD	CRO	CAZ	AES final comment										
7606.66/*Km*	OXY-5–9 (WT), CTX-M-15	ESBL	pos	pos	pos	pos	pos	pos	pos	≤4	≥8	8	≤1	ESBL (CTX-M-like)	16	15	32	8	2	22	8	11	4	28
7907.29/*Km*	OXY-1–2 (WT), CTX-M-15	ESBL	pos	pos	pos	pos	pos	pos	pos	≤4	≥8	16	4	ESBL	32	13	>32	0	8	17	16	8	16	22
5401.38/*Km*	OXY-1–2 (WT), CTX-M-15, OXA-1, TEM-1	ESBL	pos	pos	pos	pos	pos	pos	pos	16	≥8	≥64	4	ESBL	128	8	>32	0	32	12	64	0	>16	18
1312240753/*Km*	OXY-1–2 (WT), CTX-M-15, OXA-1, TEM-1	ESBL	pos	pos	pos	pos	pos	pos	pos	≥128	≥8	≥64	16	ESBL	128	8	>32	0	32	9	64	0	>16	15
8212.48/*Ko*	OXY-2–12 (WT), CTX-M-15	ESBL	pos	pos	pos	pos	pos	pos	pos	≤4	≥8	16	4	ESBL	32	13	>32	0	8	15	16	7	8	22
7407.04/*Ko*	OXY-2–16 (WT), CTX-M-15, OXA-1	ESBL	pos	pos	pos	pos	pos	pos	pos	32	≥8	≥64	≥64	ESBL	>128	0	>32	0	128	6	>64	0	>16	8
15KM0222 /*Ko*	OXY-2–7 (WT), CTX-M-1	ESBL	pos	pos	pos	pos	pos	pos	pos	≤4	≥8	16	≤1	ESBL (CTX-M-like)	>128	11	>32	0	4	19	64	7	16	21
13KM0084 /*Ko*	OXY-2–2 (WT), CTX-M-1, OXA-1	ESBL	pos	pos	pos	pos	pos	pos	pos	16	≥8	16	≤1	ESBL (CTX-M-like)	64	12	>32	0	2	20	32	8	8	24
13KM1040 /*Ko*	OXY-2–2 (WT), CTX-M-1	ESBL	pos	pos	pos	pos	pos	pos	pos	≤4	≥8	16	≤1	ESBL (CTX-M-like)	>128	12	>32	0	2	19	64	0	8	24
KM57/09/*Ko*	OXY-2–7 (WT), CTX-M-1	ESBL	pos	pos	pos	pos	pos	pos	pos	≤4	≥8	16	≤1	ESBL (CTX-M-like)	64	10	>32	0	4	19	32	7	16	20
KM24/09/*Ko*	OXY-2–7 (WT), CTX-M-1	ESBL	pos	pos	pos	pos	pos	pos	pos	≤4	≥8	16	≤1	ESBL (CTX-M-like)	>128	11	>32	0	2	20	64	7	8	22
8208.45/*Km*	OXY-1–21 (strong)	hOXY	neg	neg	neg	neg	**pos**	**pos**	neg	≥128	≥8	≥64	≤1	**ESBL (CTX-M-like); high-level K1**	16	13	8	11	2	18	2	12	>16	6
8011.16/*Km*	OXY-1–2 (strong)	hOXY	neg	neg	neg	neg	**pos**	neg	**pos**	≥128	2	8	≤1	**ESBL** **(CTX-M-like)**	8	17	4	24	0.5	21	0.5	17	>16	8
7806.19/*Km*	OXY-1–1 (strong)	hOXY	neg	neg	neg	neg	neg	neg	**pos**	≥128	1	≤1	≤1	Acquired penicillinase; IRT or OXA^*h*^	2	20	1	19	0.5	23	0.5	21	8	21
7202.30/*Km*	OXY-1–2 (strong)	hOXY	neg	neg	neg	neg	neg	neg	neg	≥128	0.5	≥64	≤1	High-level K1^*i*^	4	19	1	22	≤0.25	21	1	20	>16	15
8311.01/*Ko*	OXY-2–1 (strong)	hOXY	neg	neg	neg	neg	**pos**	neg	**pos**	≥128	4	4	≤1	**ESBL** **(CTX-M-like)**	8	15	8	15	1	25	2	18	>16	18
8309.06/*Ko*	OXY-2–32 (strong)	hOXY	neg	neg	neg	neg	**pos**	neg	**pos**	≥128	4	8	≤1	**ESBL** **(CTX-M-like)**	16	14	8	13	0.5	21	2	14	>16	9
8310.32/*Ko*	OXY-2–11 (strong)	hOXY	neg	neg	neg	neg	**pos**	neg	**pos**	≥128	2	4	≤1	**ESBL** **(CTX-M-like)**	8	17	8	14	4	25	4	17	>16	18
8310.33/*Ko*	OXY-2–11 (strong)	hOXY	neg	neg	neg	neg	**pos**	neg	**pos**	≥128	≥8	≥64	≤1	**ESBL (CTX-M-like); high-level K1**	32	11	32	8	2	21	2	12	>16	6
8306.21/*Ko*	OXY-2–32 (strong)	hOXY	neg	neg	neg	neg	**pos**	neg	**pos**	≥128	2	8	≤1	**ESBL** **(CTX-M-like)**	16	14	8	13	0.5	20	0.5	14	>16	9
8108.57/*Ko*	OXY-2–33 (strong)	hOXY	neg	neg	neg	neg	neg	neg	neg	≥128	2	≥64	≤1	**ESBL (CTX-M-like)** ^***j***^	4	19	2	18	2	24	2	19	>16	20
8111.31/*Ko*	OXY-2–12 (strong)	hOXY	neg	neg	neg	neg	**pos**	neg	**pos**	≥128	≥8	8	≤1	**ESBL** **(CTX-M-like)**	16	13	8	12	1	20	1	15	>16	7
8005.38-1/*Ko*	OXY-2–18 (strong)	hOXY	neg	neg	neg	neg	**pos**	neg	**pos**	≥128	2	4	≤1	**ESBL** **(CTX-M-like)**	8	17	8	15	1	20	1	17	>16	10
8005.38-2/*Ko*	OXY-2–18 (strong)	hOXY	neg	neg	neg	neg	**pos**	neg	**pos**	≥128	2	8	≤1	**ESBL** **(CTX-M-like)**	8	18	4	14	0.5	21	2	19	>16	11
7510.48/*Ko*	OXY-2–10 (strong)	hOXY	neg	neg	neg	neg	**pos**	neg	**pos**	≥128	2	8	≤1	**ESBL** **(CTX-M-like)**	8	16	8	14	0.5	22	2	16	>16	14
7610.07/*Ko*	OXY-2–1 (strong)	hOXY	neg	neg	neg	neg	**pos**	neg	**pos**	≥128	2	8	≤1	**ESBL** **(CTX-M-like)**	8	16	4	15	0.5	24	1	18	>16	15
7707.06/*Ko*	OXY-2–34 (strong)	hOXY	neg	neg	neg	neg	**pos**	neg	**pos**	≥128	2	4	≤1	**ESBL** **(CTX-M-like)**	8	18	4	16	1	23	4	18	>16	19
7802.78/*Ko*	OXY-2–4 (strong)	hOXY	neg	neg	neg	neg	**pos**	neg	**pos**	≥128	≥8	8	≤1	**ESBL** **(CTX-M-like)**	16	15	16	12	0.5	25	1	16	>16	15
7907.16/*Ko*	OXY-2–6 (strong)	hOXY	neg	neg	neg	neg	**pos**	neg	neg	≥128	2	2	≤1	**ESBL** **(CTX-M-like)**	8	17	4	16	1	24	1	18	>16	20
R1056/*Ko^l^*	OXY-2–14 (strong), TEM-1	hOXY	**pos**	neg	neg	neg	**pos**	**pos**	**pos**	≥128	≥8	≥64	4	**ESBL; high-level K1**	64	8	>32	0	4	16	16	9	>16	7
R1057/*Ko^l^*	OXY-2–5 (strong), TEM-1	hOXY	**pos**	**pos**	neg	neg	**pos**	**pos**	**pos**	≥128	≥8	≥64	≥64	**ESBL**	64	9	>32	0	128	6	16	8	>16	15
08KM1888 /*Ko*	OXY-2–16 (strong)	hOXY	neg	neg	neg	neg	**pos**	neg	neg	≥128	≥8	8	≤1	**ESBL** **(CTX-M-like)**	16	16	8	13	1	18	2	15	>16	8
8310.44/*Km*	OXY-1–20 (strong)	WT	nt	nt	nt	nt	nt	nt	nt	≥128	≤0.25	≤1	≤1	Acquired penicillinase	≤1	24	0.5	24	0.5	28	≤0.25	23	4	26
7507.77/*Km*	OXY-1–1 (strong)	WT	nt	nt	nt	nt	nt	nt	nt	≥128	≤0.25	≤1	≤1	Acquired penicillinase; IRT or OXA^*k*^	≤1	24	1	24	0.5	29	≤0.25	24	4	29
ZH142-C/*Km*	OXY-5–1 (WT)	WT	nt	nt	nt	nt	nt	nt	nt	≤4	≤0.25	≤1	≤1	WT penicillinase	≤1	32	≤0.25	31	≤0.25	30	≤0.25	29	≤2	38
17KM0578/*Km*	OXY-1–22 (WT), TEM-1	WT	nt	nt	nt	nt	nt	nt	nt	≤4	≤0.25	≤1	≤1	WT penicillinase	≤1	31	≤0.25	30	≤0.25	30	≤0.25	29	≤2	35
15090013 /*Km*	OXY-1–1 (WT)	WT	nt	nt	nt	nt	nt	nt	nt	≤4	≤0.25	≤1	≤1	WT penicillinase	≤1	30	≤0.25	31	≤0.25	30	≤0.25	28	≤2	35
15A0136/*Km*	OXY-1–8 (WT)	WT	nt	nt	nt	nt	nt	nt	nt	≤4	≤0.25	≤1	≤1	WT penicillinase	≤1	31	≤0.25	30	≤0.25	29	≤0.25	27	≤2	35
20M0142/*Kg*	OXY-6–4 (WT)	WT	nt	nt	nt	nt	nt	nt	nt	≤4	≤0.25	≤1	≤1	WT penicillinase	≤1	35	≤0.25	34	≤0.25	33	≤0.25	31	≤2	36
08KM1900 /*Kg*	OXY-6–4 (WT)	WT	nt	nt	nt	nt	nt	nt	nt	≤4	≤0.25	≤1	≤1	WT penicillinase	≤1	35	≤0.25	33	≤0.25	33	≤0.25	31	≤2	34
15Km1352/*Kp*	OXY-4–1 (WT)	WT	nt	nt	nt	nt	nt	nt	nt	≤4	≤0.25	≤1	≤1	WT penicillinase	≤1	35	≤0.25	31	≤0.25	31	≤0.25	29	≤2	35
17KM1096 /*Ko*	OXY-2–18 (WT)	WT	nt	nt	nt	nt	nt	nt	nt	≤4	≤0.25	≤1	≤1	WT penicillinase	≤1	36	≤0.25	32	≤0.25	31	≤0.25	28	≤2	33
14/F0005/*Ko*	OXY-2–2 (WT)	WT	nt	nt	nt	nt	nt	nt	nt	≤4	≤0.25	≤1	≤1	WT penicillinase	≤1	30	≤0.25	30	≤0.25	31	≤0.25	28	≤2	32
09KM0284 /*Ko*	OXY-2–4 (WT), TEM-1	WT	nt	nt	nt	nt	nt	nt	nt	≤4	≤0.25	≤1	≤1	WT penicillinase	≤1	30	≤0.25	30	≤0.25	32	≤0.25	30	≤2	33

^*a*^ Note: *Km*, *K. michiganensis*; *Kg*, *K. grimontii*; PTZ, piperacillin-tazobactam; IRT, inhibitor resistant TEM; nt, not tested (i.e., confirmatory ESBL tests performed only when CRO MIC >1 µg/mL).

^*b*^ Full data (e.g., epidemiological data, ST, ARGs) in File S1 and reference 3.

^*c*^ Species confirmed by WGS and implementation of the Type Strain Genome Server.

^*d*^ Based on the BMD results obtained with the ESB1F/GNX2F Sensititre panels (i.e., hOXY defined as strains with ceftriaxone MIC >1 µg/mL and not possessing ESBL and/or pAmpCs encoding genes). Full MIC results are available in File S1.

^*e*^ Results highlighted in bold indicate false-positive results compared to the gold standard (i.e., WGS).

^*f*^ The DDST was performed according to EUCAST: CTX (5 µg), CAZ (10 µg), FEP (30 µg), and aztreonam (30 µg) around a disk of AMC (20-10 µg) (18).

^*g*^ Strains were provided to the Laboratory for Clinical Microbiology of IFIK with coded names. AST results of VITEK 2 showed that all strains had ertapenem MICs ≤0.5 µg/mL, imipenem MICs ≤0.5 µg/mL, and meropenem MICs ≤0.25 µg/mL. Full AST results are shown in File S1.

^*h*^ VITEK 2 repeated two times with identical results.

^*i*^ VITEK 2 repeated two times: first test with CRO MIC ≤1 µg/mL and AES with “Acquired penicillinase; IRT or OXA.” The second assay is reported in the present table.

^*j*^ VITEK 2 repeated two times: the first test was inconsistent. The second assay is reported in the present table.

^*k*^ VITEK 2 repeated two times: first test with AES with “Acquired penicillinase.” The second assay is reported in the present table.

^*l*^ These two strains were isolated from the same patient (R1057 after prolonged treatment with ceftazidime). OXY-2–5 hydrolyzes CAZ at much higher level than other OXY variants (5).

^*m*^ Tested two times at the IFIK and once at the APL.

Our strains showed features consistent with those reported in other contemporary studies (i.e., most of the ESBL producers possessed *bla*_CTX-Ms_) (7–13). However, two hOXY-*Ko*C isolates (R1056 and R1057) produced uncommon OXY enzyme variants capable of hydrolyzing very well CAZ and, therefore, conferring a true ESBL-like spectrum of activity (5). Overall, this well-defined collection of *Ko*C strains, including its total number, is unparalleled in the context of previous studies focusing on the recognition of ESBL producers using standard (20, 24, 30–34) or automated methods such as VITEK 2 (23–27, 29, 35–38). We believe that these attributes contribute to the robustness and illustrative value of the present analysis.

### VITEK 2 system with the AES

All 11 ESBL-*Ko*C strains were correctly identified as ESBL producers by VITEK 2 in conjunction with the AES (Table 1). In particular, strains producing CTX-M-1 were indicated as “ESBL (CTX-M-like),” while those with CTX-M-15 as generic “ESBL” (with the exception of strain 7606.66). We speculate that this distinction was probably based on the CAZ MIC (i.e., ≥4 µg/mL for CTX-M-15 and ≤1 µg/mL for CTX-M-1 producers).

In contrast, almost all hOXY-*Ko*C (19/21; 90.5%) were erroneously identified as ESBL producers. Only one hOXY-*Ko*C was correctly reported as “high-level K1” (strain 7202.30), while three more strains were also reported with this AES comment, but together with “ESBL” or “ESBL/(CTX-M-like).” Moreover, three *Ko*C strains were indicated as producers of “IRT or OXA” and/or “acquired penicillinase,” but no corresponding encoding genes for these enzymes were detected by using the WGS output (Table 1).

Overall, the VITEK AST-N242 card with the enabled AES showed 100% Se and 64.7% Sp for the detection of ESBL-*Ko*C strains. This low Sp was due to the many false positives (FPs) in the group of hOXY strains, a result consistent with that observed in our previous study for some of the ESBL-CTs such as EUCAST DDST-25, BMD with CTX/CTX-CL, and gradient strip with FEP/FEP-CL (Sp of 66.0%, 64.7%, and 67.3%, respectively) (Table 1; File S1) (3). We emphasize that the erroneous detection of ESBL producers may imply unnecessary isolation of carriers for preventing outbreaks and, consequently, increased health-care costs. Moreover, clinicians may also be more prone to use carbapenems for therapy instead of third-/fourth-generation cephalosporins (e.g., CAZ), even when the latter fall within the susceptible range.

As anticipated, a direct comparison with previous studies where the VITEK 2/AES performance was evaluated to detect ESBL-*Ko*C strains is difficult. These investigations were conducted more than 13 years ago and included a mixture of Enterobacterales (mainly *E. coli* and *K. pneumoniae*) in which *Ko* strains were present in very low numbers. Moreover, the molecular typing of such *Ko* strains was limited (e.g., both *bla*_OXYs_ and their promoter regions were not fully characterized) when compared to the present work (Table 1) (23–27, 35–38). Finally, several other studies that included *Ko* information were performed using VITEK cards that provided the ESBL test (discontinued by the manufacturer), which was an additional parameter interpreted by the AES (39). In this regard, using the AES, three studies reported FP ESBL-*Ko*C strains that were actually only OXY producers by molecular analyses (i.e., 1 out of 2, 3 out of 9, and 1 out of 21, respectively) (25, 35, 36). Furthermore, both Robin et al. and Spanu et al. obtained 100% Se and 100% Sp when testing their collection of *Ko* strains (*n* = 3, of which two were true ESBL producers; *n* = 38, of which two were true ESBL producers, respectively) (37, 38).

As in the present study, several earlier studies were also done using the AES and VITEK cards not equipped with the ESBL test. However, after extrapolating their specific *Ko* results, no correlation with our high number of FP ESBL producers was noted. In fact, these analyses indicated that the number of FP ESBL-*Ko* strains was very low or null (23–26), although this approach compromised the detection of about half of the true ESBL-*Ko* strains (23–25, 27). For instance, Wiegand et al. tested 16 *Ko* strains of which five were true ESBL producers: the VITEK 2/AES reported one FP and two false-negative (FN) ESBL producers (71.4% Se and 90.0% Sp) (24). In another study, Nyberg et al. evaluated 25 *Ko* strains of which four were true ESBL producers: the AES reported two FPs and three FNs (57.1% Se and 91.3% Sp) (25).

It is therefore not clear why the VITEK 2/AES assessment at the IFIK showed a very high FP ESBL rate for *Ko*C strains that were only hOXY (Table 1). It could be speculated that our contemporary collection of *Ko*C strains produces OXYs enzymes with characteristics not yet considered by the AES database. We also hypothesized that the EUCAST criteria used by our AES may somehow negatively affect the interpretative algorithms set for *Ko* strains (18, 28). In this context, we noted that all previous VITEK 2 evaluations using cards without the ESBL test employed the CLSI criteria published between 2000 and 2007 (23–26).

Based on the above observations, we believe it is justified that the AES should be disabled when testing *Ko*C isolates of clinical importance. Moreover, once the VITEK 2 ASTs are available, *Ko*C strains suspicious for ESBL production should be tested with ESBL-CTs that demonstrated high Sp such as the CDTs (Table 1) (3). However, for the purpose of this study, disabling the AES at IFIK would cause significant disruption to daily routine testing. It would also require formal intervention from the company to modify the software and adjust all internal standard operating procedures. In contrast, these complex and time-consuming changes could be effectively avoided by submitting the strains to another independent clinical institution that does not routinely implement the AES. To do so, all 44 *Ko*C strains were blindly submitted to the APL (see below).

### VITEK 2 system without AES

At the APL, the blinded 44 *Ko*C strains underwent VITEK 2 ASTs followed by CLSI CDTs when the resulting CRO and/or CAZ MICs were >1 µg/mL. As a result, considering the output of the two CDTs for a total of 33 *Ko*C strains tested (11 ESBL producers, 21 hOXY, and 1 WT), the overall approach demonstrated 100% Se and 95.7% Sp in detecting ESBL producers (Table 2). This was a remarkable superior analytic performance compared to that of the VITEK 2/AST-N242 card coupled by the AES conducted at the IFIK (100% Se and 64.7% Sp for the overall 44 *Ko*C strains; 100% Se and 53.7% Sp for the above subgroup of 33 strains). The results of the VITEK 2 system without AES combined with CLSI screening and ESBL-CTs were also superior to most of the studies performed in the past (23–27, 35–38).

**TABLE 2:** Results of the VITEK 2 ASTs and ESBL-CTs obtained at the APL (Calgary, Canada)^*a*^

Strain/species^*b*^^,^^*c*^	*Ko*C group^*d*^	VITEK AST–N390 card (MIC, µg/mL) without the Advanced Expert System^*e*^				CLSI CDTs (based on VITEK 2 CRO/CAZ MICs)^*f*^			CAZ 30 µg disk (mm)	CTX 30 µg disk (mm)	CFM 5 µg disk (mm)
		PTZ	CFM	CRO	CAZ	CTX / CTX-CL	CAZ / CAZ-CL	Final report			
7606.66/*Km*	ESBL	≤4	1	32	0.5	pos	**neg**	ESBL	28	20	21
7907.29/*Km*	ESBL	≤4	≥4	32	4	pos	**pos**	ESBL	23	15	16
5401.38/*Km*	ESBL	16	≥4	≥64	8	pos	pos	ESBL	20	9	9
1312240753/*Km*	ESBL	≥128	≥4	≥64	32	pos	pos	ESBL	15	10	6
8212.48/*Ko*	ESBL	≤4	≥4	32	4	pos	pos	ESBL	22	15	15
7407.04/*Ko*	ESBL	32	≥4	≥64	≥64	pos	pos	ESBL	8	6	6
15KM0222/*Ko*	ESBL	≤4	2	≥64	1	pos	**neg**	ESBL	25	13	20
13KM0084/*Ko*	ESBL	16	0.5	32	0.5	pos	**neg**	ESBL	29	13	24
13KM1040/*Ko*	ESBL	≤ 4	0.5	32	0.5	pos	**neg**	ESBL	25	13	23
KM57/09/*Ko*	ESBL	≤ 4	2	≥ 64	16	pos	**neg**	ESBL	24	13	18
KM24/09/*Ko*	ESBL	≤ 4	0.5	≥ 64	4	pos	**neg**	ESBL	24	14	23
8208.45/*Km*	hOXY	≥128	≥4	32	4	neg	neg	hOXY	23	24	20
8011.16/*Km*	hOXY	≥128	1	32	4	neg	neg	hOXY	25	23	20
7806.19/*Km*	hOXY	≥128	≤0.25	16	0.25	neg	neg	hOXY	24	26	27
7202.30/*Km*	hOXY	≥128	≤0.25	8	0.25	neg	neg	hOXY	29	30	27
8311.01/*Ko*	hOXY	≥128	≤0.25	4	≤0.12	neg	neg	hOXY	30	28	30
8309.06/*Ko*	hOXY	≥128	0.5	32	0.5	neg	neg	hOXY	25	25	23
8310.32/*Ko*	hOXY	≥128	≤0.25	4	≤0.12	neg	neg	hOXY	29	28	28
8310.33/*Ko*	hOXY	≥128	≤0.25	8	≤0.12	neg	neg	hOXY	28	23	22
8306.21/*Ko*	hOXY	≥128	≤0.25	32	0.5	neg	neg	hOXY	25	26	23
8108.57/*Ko*	hOXY	64	≤0.25	8	≤0.12	neg	neg	hOXY	30	32	30
8111.31/*Ko*	hOXY	≥128	0.5	32	1	neg	neg	hOXY	24	26	21
8005.38–1/*Ko*	hOXY	≥128	≤0.25	8	≤0.12	neg	neg	hOXY	30	30	29
8005.38–2/*Ko*	hOXY	≥128	≤0.25	8	0.5	neg	neg	hOXY	25	28	25
7510.48/*Ko*	hOXY	≥128	≤0.25	32	≤0.12	neg	neg	hOXY	28	28	27
7610.07/*Ko*	hOXY	≥128	≤0.25	32	≤0.12	neg	neg	hOXY	28	28	27
7707.06/*Ko*	hOXY	≥128	≤0.25	8	≤0.12	neg	neg	hOXY	30	29	28
7802.78/*Ko*	hOXY	≥128	≤0.25	8	0.25	neg	neg	hOXY	30	28	28
7907.16/*Ko*	hOXY	≥128	≤0.25	8	≤0.12	neg	neg	hOXY	32	30	29
R1056/*Ko*	hOXY	≥128	≥ 4	≥ 64	4	neg	neg	hOXY	21	17	13
R1057/*Ko^g^*	hOXY	32	≥ 4	≥ 64	≥ 64	neg	**pos**	**ESBL**	6	16	7
08KM1888/*Ko*	hOXY	≥128	0.5	32	2	neg	neg	hOXY	26	25	25
8310.44/*Km*	WT	≥128	≤0.25	0.5	≤0.12	–	–	–	30	32	29
7507.77/*Km*	WT	≥128	≤0.25	8	≤0.12	neg	neg	**hOXY**	30	30	30
ZH142-C / *Km*	WT	≤4	≤0.25	≤0.25	≤0.12	–	–	–	32	31	33
17KM0578/*Km*	WT	≤4	≤0.25	≤0.25	≤0.12	–	–	–	29	32	32
15090013/*Km*	WT	≤4	≤0.25	≤0.25	≤0.12	–	–	–	31	30	32
15A0136/*Km*	WT	≤4	≤0.25	≤0.25	≤0.12	–	–	–	32	31	30
20M0142/*Kg*	WT	≤4	≤0.25	≤0.25	≤0.12	–	–	–	30	32	34
08KM1900/*Kg*	WT	≤4	≤0.25	≤0.25	≤0.12	–	–	–	29	31	32
15Km1352/*Kp*	WT	≤4	≤0.25	≤0.25	≤0.12	–	–	–	32	30	32
17KM1096/*Ko*	WT	≤4	≤0.25	≤0.25	≤0.12	–	–	–	31	32	33
14/F0005 / *Ko*	WT	≤4	≤0.25	≤0.25	≤0.12	–	–	–	30	32	31
09KM0284/*Ko*	WT	≤4	≤0.25	≤0.25	≤0.12	–	–	–	30	32	33

^*a*^ Note: *Km*, *K. michiganensis*; *Kg*, *K. grimontii*; PTZ, piperacillin–tazobactam; CFM, cefixime; ESBL, ESBL producer; –, not tested or not applicable.

^*b*^ Species confirmed by WGS and implementation of the Type Strain Genome Server.

^*c*^ Full molecular characterization reported in File S1 and in reference 3.

^*d*^ Based on the BMD results obtained with the ESB1F/GNX2F Sensititre panels (i.e., hOXY defined as strains with CRO MIC >1 µg/mL and not possessing ESBL and/or pAmpCs encoding genes). See Table 1.

^*e*^ Assays performed at the APL. Strains were provided by the IFIK with coded names. AST results of VITEK 2 showed that all strains had ertapenem MICs ≤0.12 µg/mL and meropenem MICs ≤0.25 µg/mL (full AST results in File S1).

^*f*^ CDTs were implemented when the VITEK 2 MIC for CRO/CAZ were >1 µg/mL. Results highlighted in bold indicate FN or FP results compared to the gold standard (i.e., WGS). Full results of the CLSI CDTs are shown in File S1.

^*g*^ VITEK 2 and CLSI CDTs repeated two times with consistent results.

This part of the study also highlighted two important aspects. First, a screening CRO and/or CAZ MIC cutoff of >1 µg/mL (EUCAST/CLSI criteria) implied a high number of unnecessary ESBL-CTs for *Ko*C strains (*n* = 22), which mainly belonged to the hOXY group (*n* = 21) (Table 2) (18, 19).

Second, the CLSI-recommended CDT with CAZ/CAZ-CL (30/30–10 µg disks) had an inferior performance when compared to the corresponding EUCAST assay (10/10–10 µg disks) testing the same 33 *Ko*C strains (i.e., 64.7% Se and 95.7% Sp *vs.* 100% Se and 95.7% Sp, respectively) (Tables 1 and 2). On this matter, studies that compared the EUCAST- and CLSI-recommended CDTs to distinguish between ESBL-*Ko*C and hOXY-*Ko*C had not been previously performed. Nevertheless, testing Enterobacterales (*n* = 236 of which 17 were *Ko*), Polsfuss et al. also showed that the disk approximation method with the EUCAST 10 µg CAZ disk detected slightly more ESBL producers than when using the CLSI CAZ 30 µg disk (i.e., 86.4% Se and 98.3% Sp *vs.* 84.7% Se and 98.3% Sp, respectively) (40).

### Screening cutoffs to perform ESBL-CTs

In order to limit the use of superfluous ESBL-CTs for *Ko*C isolates in routine clinical laboratories, we analyzed the MICs obtained in BMD or with VITEK 2 for CRO, CPD, CAZ, CTX, and ATM against our 44 strains. Furthermore, the same antibiotics were also tested by disk diffusion according to the EUCAST and CLSI criteria (Tables 1 and 2). Results of each method and drug were stratified in cutoffs and depicted in Table 3.

**TABLE 3:** Exploring ceftriaxone, cefpodoxime, ceftazidime, cefotaxime, and aztreonam cutoffs to limit the use of unnecessary ESBL-CTs for *Ko*C strains^*a*^

Screening cutoff for *Ko*C	No. (%) of ESBL confirmatory tests performed with the cutoff^*b*^				Screening performance of the cutoff			
	ESBL producers (*n* = 11)	hOXY (*n* = 21)	WT (*n* = 12)	Total *Ko*C tested (*n* = 44)	Se	Sp	PPV	NPV
Ceftriaxone (CRO)								
MIC in BMD (Sensititre)^*c*^								
>1 µg/mL (EUCAST/CLSI cutoff)	11 (100%)	21 (100%)	–	32 (72.7%)	100%	61.1%	34.4%	100%
>2 µg/mL	11 (100%)	20 (95.2%)	–	31 (70.5%)	100%	62.3%	35.5%	100%
>4 µg/mL	11 (100%)	18 (85.7%)	–	29 (65.9%)	100%	64.7%	37.9%	100%
>8 µg/mL^*d*^	11 (100%)	9 (42.9%)	–	20 (45.5%)	100%	78.6%	55.0%	100%
>16 µg/mL	10 (90.9%)	3 (14.3%)	–	13 (29.5%)	91.7%	91.7%	78.6%	97.1%
MIC with VITEK 2 AST– N242 card^*c*^								
>1 µg/mL (EUCAST/CLSI cutoff)	11 (100%)	20 (95.2%)	–	31 (70.5%)	100%	62.3%	35.5%	100%
>2 µg/mL	11 (100%)	19 (90.5%)	–	30 (68.2%)	100%	63.5%	36.7%	100%
>4 µg/mL^*d*^	11 (100%)	15 (71.4%)	–	26 (59.1%)	100%	68.8%	42.3%	100%
>8 µg/mL	10 (90.9%)	6 (28.6%)	–	16 (36.4%)	91.7%	84.6%	64.7%	97.1%
MIC with VITEK 2 AST-N390 card^*e*^								
>0.5 µg/mL	11 (100%)	21 (100%)	1 (8.3%)	33 (75.0%)	100%	60.0%	33.3%	100%
>1 µg/mL (EUCAST/CLSI cutoff)	11 (100%)	21 (100%)	1 (8.3%)	33 (75.0%)	100%	60.0%	33.3%	100%
>2 µg/mL	11 (100%)	21 (100%)	1 (8.3%)	33 (75.0%)	100%	60.0%	33.3%	100%
>4 µg/mL	11 (100%)	19 (90.5%)	1 (8.3%)	31 (70.5%)	100%	62.3%	35.5%	100%
>8 µg/mL	11 (100%)	11 (52.4%)	–	22 (50.0%)	100%	75.0%	50.0%	100%
>16 µg/mL^*d*^	11 (100%)	10 (47.6%)	–	21 (47.7%)	100%	76.7%	52.4%	100%
>32 µg/mL	6 (54.5%)	2 (6.5%)	–	8 (18.2%)	68.8%	94.3%	84.6%	97.1%
EUCAST/CLSI 30 µg disk (inhibition zone)^*c*^^,^^*f*^								
≤25 mm (CLSI cutoff)	11 (100%)	21 (100%)	2 (16.7%)	34 (77.3%)	100%	58.9%	33.3%	100%
≤22 mm (EUCAST cutoff)	11 (100%)	21 (100%)	–	33 (75.0%)	100%	61.1%	33.3%	100%
≤16 mm	11 (100%)	12 (57.1%)	–	23 (52.3%)	100%	73.3%	47.8%	100%
≤15 mm^*d*^	11 (100%)	9 (42.9%)	–	20 (45.5%)	100%	78.6%	55.0%	100%
≤14 mm	10 (90.9%)	7 (33.3%)	–	17 (38.6%)	91.7%	82.5%	61.1%	97.1%
Cefpodoxime (CPD)								
MIC in BMD (Sensititre)^*c*^								
>1 µg/mL (EUCAST cutoff)	11 (100%)	19 (90.5%)	–	30 (68.2%)	100%	63.5%	36.7%	100%
>2 µg/mL	11 (100%)	18 (85.7%)	–	29 (65.9%)	100%	64.7%	37.9%	100%
>4 µg/mL (CLSI cutoff)	11 (100%)	13 (61.9%)	–	24 (54.5%)	100%	71.7%	45.8%	100%
>8 µg/mL	11 (100%)	4 (19.0%)	–	15 (34.1%)	100%	89.2%	73.3%	100%
>16 µg/mL^*d*^	11 (100%)	3 (14.3%)	–	14 (31.8%)	100%	91.7%	78.6%	100%
>32 µg/mL	10 (90.9%)	2 (6.5%)	–	12 (27.3%)	91.7%	94.3%	84.6%	97.1%
MIC with VITEK 2 AST-N242 card^*e*^								
>1 µg/mL (EUCAST cutoff)	11 (100%)	19 (90.5%)	–	30 (68.2%)	100%	63.5%	36.7%	100%
>2 µg/mL	11 (100%)	9 (42.9%)	–	20 (45.5%)	100%	78.6%	55.0%	100%
>4 µg/mL (CLSI cutoff)	11 (100%)	7 (33.3%)	–	18 (40.9%)	100%	82.5%	61.1%	100%
≥8 µg/mL^*d*^	11 (100%)	7 (33.3%)	–	18 (40.9%)	100%	82.5%	61.1%	100%
EUCAST/CLSI 10 µg disk (inhibition zone)^*c*^^,^^*f*^								
≤20 mm (EUCAST cutoff)	11 (100%)	19 (90.5%)	–	30 (68.2%)	100%	63.5%	36.7%	100%
≤17 mm (CLSI cutoff)	11 (100%)	17 (81.0%)	–	28 (63.6%)	100%	66.0%	39.3%	100%
≤12 mm	11 (100%)	6 (28.6%)	–	17 (38.6%)	100%	84.6%	64.7%	100%
≤10 mm	11 (100%)	3 (14.3%)	–	14 (31.8%)	100%	91.7%	78.6%	100%
≤8 mm^*d*^	11 (100%)	2 (6.5%)	–	13 (29.5%)	100%	94.3%	84.6%	100%
≤7 mm	10 (90.9%)	2 (6.5%)	–	12 (27.3%)	91.7%	94.3%	84.6%	97.1%
Ceftazidime (CAZ)								
MIC in BMD (Sensititre)^*c*^								
>0.5 µg/mL	11 (100%)	12 (57.1%)	–	23 (52.3%)	100%	73.3%	47.8%	100%
>1 µg/mL (EUCAST/CLSI cutoff)^*d*^	11 (100%)	6 (28.6%)	–	17 (38.6%)	100%	84.6%	64.7%	100%
>2 µg/mL	7 (63.6%)	3 (14.3%)	–	10 (22.7%)	73.3%	91.7%	78.6%	89.2%
MIC with VITEK 2 AST– N242 card^*c*^								
>1 µg/mL (EUCAST/CLSI cutoff)	5 (45.5%)	2 (6.5%)	–	7 (15.9%)	64.7%	94.3%	84.6%	84.6%
MIC with VITEK 2 AST-N390 card^*e*^								
>0.5 µg/mL	8 (72.7%)	6 (28.6%)	–	14 (31.8%)	78.6%	84.6%	64.7%	91.7%
>1 µg/mL (EUCAST/CLSI cutoff)	7 (63.6%)	5 (23.8%)	–	12 (27.3%)	73.3%	86.8%	68.8%	89.2%
EUCAST 10 µg disk (inhibition zone)^*c*^								
≤23 mm	11 (100%)	15 (71.4%)	–	26 (59.1%)	100%	68.8%	42.3%	100%
≤22 mm^*d*^	11 (100%)	13 (61.9%)	–	24 (54.5%)	100%	71.7%	45.8%	100%
≤21 mm (EUCAST cutoff)	10 (90.9%)	12 (57.1%)	–	22 (50.0%)	91.7%	73.3%	47.8%	97.1%
CLSI 30 µg disk (inhibition zone)^*e*^^,^^*f*^								
≤30 mm	11 (100%)	20 (95.2%)	6 (50.0%)	37 (84.1%)	100%	55.9%	29.7%	100%
≤29 mm^*d*^	11 (100%)	15 (71.4%)	2 (16.7%)	28 (63.6%)	100%	66.0%	39.3%	100%
≤28 mm	10 (90.9%)	13 (61.9%)	–	23 (52.3%)	91.7%	71.7%	45.8%	97.1%
≤26 mm	9 (81.8%)	10 (47.6%)	–	19 (43.2%)	84.6%	76.7%	52.4%	94.3%
≤24 mm	7 (63.6%)	5 (23.8%)	–	12 (27.3%)	73.3%	86.8%	68.8%	89.2%
≤22 mm (CLSI cutoff)	4 (36.4%)	2 (6.5%)	–	6 (13.6%)	61.1%	94.3%	84.6%	82.5%
Cefotaxime (CTX)								
MIC in BMD (Sensititre)^*c*^								
>1 µg/mL (EUCAST/CLSI cutoff)	11 (100%)	12 (57.1%)	–	23 (52.3%)	100%	73.3%	47.8%	100%
>2 µg/mL	11 (100%)	4 (19.0%)	–	15 (34.1%)	100%	89.2%	73.3%	100%
>4 µg/mL^*d*^	11 (100%)	2 (6.5%)	–	13 (29.5%)	100%	94.3%	84.6%	100%
>8 µg/mL	10 (90.9%)	2 (6.5%)	–	12 (27.3%)	91.7%	94.3%	84.6%	97.1%
EUCAST 5 µg disk (inhibition zone)^*c*^^,^^*f*^								
≤20 mm (EUCAST cutoff)	11 (100%)	20 (95.2%)	–	31 (70.5%)	100%	62.3%	35.5%	100%
≤16 mm	11 (100%)	10 (47.6%)	–	21 (47.7%)	100%	76.7%	52.4%	100%
≤12 mm	11 (100%)	4 (19.0%)	–	15 (34.1%)	100%	89.2%	73.3%	100%
≤11 mm^*d*^	11 (100%)	2 (6.5%)	–	13 (29.5%)	100%	94.3%	84.6%	100%
≤10 mm	10 (90.9%)	2 (6.5%)	–	12 (27.3%)	91.7%	94.3%	84.6%	97.1%
CLSI 30 µg disk (inhibition zone)^*e*^^,^^*f*^								
≤27 mm (CLSI cutoff)	11 (100%)	9 (42.9%)	–	20 (45.5%)	100%	78.6%	55.0%	100%
≤22 mm	11 (100%)	2 (6.5%)	–	13 (29.5%)	100%	94.3%	84.6%	100%
≤20 mm^*d*^	11 (100%)	2 (6.5%)	–	13 (29.5%)	100%	94.3%	84.6%	100%
≤19 mm	10 (90.9%)	2 (6.5%)	–	12 (27.3%)	91.7%	94.3%	84.6%	97.1%
Aztreonam (ATM)								
MIC in BMD (Sensititre)^*c*^								
>1 µg/mL (CLSI cutoff)	11 (100%)	21 (100%)	2 (16.7%)	34 (77.3%)	100%	58.9%	32.4%	100%
>2 µg/mL	11 (100%)	21 (100%)	2 (16.7%)	34 (77.3%)	100%	58.9%	32.4%	100%
>4 µg/mL^*d*^	10 (90.9%)	21 (100%)	–	31 (70.5%)	100%	61.1%	34.4%	97.1%
EUCAST/CLSI 30 µg disk (inhibition zone)^*c*^								
≤28 mm	11 (100%)	21 (100%)	1 (8.3%)	33 (75.0%)	100%	60.0%	33.3%	100%
≤27 mm (CLSI cutoff)^*d*^	10 (90.9%)	21 (100%)	1 (8.3%)	32 (72.7%)	91.7%	60.0%	33.3%	97.1%

^*a*^ Note: –, zero.

^*b*^ Best performance can be obtained with CDT FEP/FEP–CL, EUCAST DDST at 30 mm, or CDTs CTX/CTX–CL and CAZ/CAZ–CL together (EUCAST or CLSI). See Tables 1 and 2.

^*c*^ See full results in Table 1 and/or File S1.

^*d*^ Last cutoff to include all 11 ESBL producers (i.e., 100% Se).

^*e*^ See full results in Table 2.

^*f*^ Inhibition zone cutoff were shown in a non–linear way to save space, but without missing essential information about Se and Sp.

Based on our data, we proposed alternative screening cutoffs to perform the ESBL-CTs for *Ko*C strains (Table 4). To do so, we also considered previous investigations from which data about MICs or inhibition zone diameters for *Ko*C strains was available (File S1). Notably, we defined the possible *Ko*C cutoffs based on the following aspects. First, the screening cutoffs were selected to guarantee 100% Se and a higher Sp/PPV. Second, conservative cutoffs for the disk diffusion method were designated to avoid possible subjective reading variances (e.g., ±1 mm). Third, we considered the differences between the BMD and VITEK 2 methods (i.e., in terms of results obtained and MIC ranges tested (File S2) to propose homogeneous cutoffs for each cephalosporin and ATM.

**TABLE 4:** Summary of the ESBL screening cutoff performance for our collection of 44 *Ko*C strains: present EUCAST/CLSI criteria *versus* proposed cutoffs^*a*^

Screening criterion	ESBL screening cutoffs									
	CRO		CPD		CAZ		CTX		ATM^*j*^	
	MIC (µg/mL)	Inhibition zone (mm)^*b*^	MIC (µg/mL)	Inhibition zone (mm)^*c*^	MIC (µg/mL)	Inhibition zone (mm)^*d*^	MIC (µg/mL)	Inhibition zone (mm)^*e*^	MIC (µg/mL)	Inhibition zone (mm)
EUCAST 2024 (18) Se/Sp	>1 BMD: 100%/61.1% VITEK 2: 100%/60.0–62.3%^*h*^	≤22 100%/61.1%	>1 BMD: 100%/63.5% VITEK 2: 100%/63.5%	≤20 100%/63.5%	>1 BMD: 100%/84.6% VITEK 2: 64.7–73.3%/86.8–94.3%^*h*^	≤21 91.7%/73.3**%**	>1 BMD: 100%/73.3% VITEK 2: –	≤20 100%/62.3%	**–**	**–**
CLSI 2024 (19) Se/Sp		≤25 100%/58.9%	>4 BMD: 100%/71.7% VITEK 2: 100%/82.5%	≤17 100%/66.0%		≤22 61.1%/94.3%		≤27 100%/78.6%	**>1** BMD: 100%/58.9% VITEK 2: –	≤27 91.7%/60.0%
Proposed cutoff for *Ko*C strains^*f*^	>4	≤16	>4	≤10	>1	EUCAST disk: ≤22 CLSI disk: ≤30	>4	EUCAST disk: ≤12 CLSI disk: ≤22	>1	≤28
Se/Sp	BMD: 100%/64.7% VITEK 2: 100%/62.3–68.8%^*h*^	100%/73.3%	Same of CLSI	100%/91.7%	–	EUCAST disk: 100%/71.7% CLSI disk: 100%/55.9%	BMD: 100%/94.3%	EUCAST disk: 100%/89.2% CLSI disk: 100%/94.3%	–	100%/60.0%
Saved ESBL confirmatory tests^*g*^	BMD: 6.8% VITEK 2: 4.5–11.4%^*h*^	EUCAST: 22.7% CLSI: 25.0%	BMD: 14.0% VITEK 2: 27.3%^*i*^	EUCAST: 36.4% CLSI: 31.8%	–	EUCAST disk: 13.8% more CLSI disk: 70.5% more	BMD: 22.8%	EUCAST disk: 36.4% CLSI disk: 16.0%	–	2.3%

^*a*^ –, not available or not applicable; Se, sensitivity; Sp, specificity; BMD, broth microdilution.

^*b*^ EUCAST and CLSI suggest the use of the same 30 µg disk.

^*c*^ EUCAST and CLSI suggest the use of the same 10 µg disk.

^*d*^ EUCAST suggest the use of 10 µg disk, whereas CLSI one of 30 µg disk.

^*e*^ EUCAST suggest the use of 5 µg disk, whereas CLSI one of 30 µg disk.

^*f*^ Cutoff set to assure 100% sensitivity to screen for ESBL producers. We also considered technical differences and the literature (see text).

^*g*^ Compared to the current EUCAST and CLSI screening criteria.

^*h*^ Range between the two VITEK cards used (AST-N242 and AST-N390).

^*i*^ *vs* EUCAST only.

^*j*^ Suggested only by CLSI (30 µg disk).

### Screening cutoffs using CRO

As shown in Table 3, implementation of the common CRO EUCAST/CLSI cutoff (MIC >1 µg/mL) with the BMD method assured 100% Se, but only 61.1% Sp and 34.4% PPV when tested against our 44 *Ko*C strains. In contrast, a cutoff of >8 µg/mL could retain 100% Se, but increasing Sp and PPV to 78.6% and 55%, respectively. We also noted that other authors reported CRO MICs for the CTX-M-producing *Ko*C strains of ≥8 µg/mL, whereas those of the non-ESBL-positive strains were in great part <4 µg/mL (File S2) (23, 34, 41). Finally, a cutoff of >8 µg/mL for the screening of *Enterobacteriaceae* was also suggested by Huang et al. (42).

Nevertheless, a CRO cutoff of >8 µg/mL was appropriate only for the VITEK AST-N390 card, whereas for the AST-N242 card, the best cutoff was >4 µg/mL (Table 3). Therefore, we propose that the CRO MIC ESBL screening cutoff for *Ko*C strains should be set at >4 µg/mL. According to our data, this conservative cutoff may assure 100% Se, ~62%–69% Sp, and less performed ESBL-CTs (~7%–11%) when compared to the current EUCAST/CLSI criteria (Table 4).

Concerning the disk diffusion method, EUCAST and CLSI suggest CRO screening cutoffs for the 30 µg disk of ≤22 mm and ≤25 mm, respectively (18, 19). For our collection, these cutoffs demonstrated 100% Se, but low Sp (~59%–61%) and PPV (33%). In contrast, a cutoff of ≤15 mm could have guaranteed 100% Se, 78.6% Sp, and 55% PPV (Table 3).

Unfortunately, data regarding *Ko*C strains and disk diffusion susceptibility such as that of CRO is lacking. Only Fujita et al. reported that two strains producing CTX-Ms had CRO inhibition diameters of 10 and 12 mm (File S2) (17). Consequently, we advise a cautionary cutoff of ≤16 mm that may guarantee 100% Se, 73.3% Sp, and 47.8% PPV for *Ko*C strains (also saving ~25% of the superfluous ESBL-CTs) (Table 4).

### Screening cutoffs using CPD

The MIC screening cutoffs for CPD set by EUCAST and CLSI differ substantially (>1 and >4 µg/mL, respectively) (19, 28). By implementing the BMD method with our collection of 44 *Ko*C strains, both limits assured 100% Se, but low Sp (~64%–72%) and PPV (~37%–46%). On the other hand, a cutoff of >16 µg/mL could offer 100% Se, 91.7% Sp, and 78.6% PPV (Table 3). Of note, previous surveys involving ESBL- and hOXY-*Ko*C strains have shown CPD MICs of ≥8 µg/mL (range: 8 to >64 µg/mL) and ≤4 µg/mL (range: 1 to 4 µg/mL), respectively (File S2) (23, 34).

Nevertheless, since the highest concentration tested by the VITEK AST-N242 card is 8 µg/mL, we suggest an ESBL screening cutoff for CPD of >4 µg/mL for *Ko*C strains (Table 3). This cutoff is identical to that currently set by CLSI, but it may save an additional ~14%–27% of the confirmatory tests for *Ko*C strains if also applied by EUCAST (Table 4).

EUCAST and CLSI indicate CPD screening cutoffs for the 10 µg disk of ≤20 mm and ≤17 mm, respectively (18, 19). Our analysis showed that these cutoffs generated 100% Se, but low Sp (~64%–66%) and PPV (~37%–39%). In contrast, a limit of ≤8 mm could have assured 100% Se, 94.3% Sp, and 84.6% PPV (Table 3). In this context, Fujita et al. noted that the CPD inhibition diameters for the two CTX-M producers were ≤8 mm, while those for non-ESBL-positive strains were ≥12 mm in 92% of the cases (File S2) (17). As a result, we propose that a CPD screening cutoff of ≤10 mm may be appropriate when testing *Ko*C strains. This lower limit may assure 100% Se, 91.7% Sp, 78.6% PPV, and a reduction of the ESBL-CTs of ~32%–36% when compared to the current EUCAST and CLSI screening criteria (Table 4).

### Screening cutoffs using CAZ

In the past, CAZ had been indicated as the key substrate to distinguish ESBL- from hOXY-*Ko*C strains (31, 32). This indication was based on the observation that WT OXY enzymes hydrolyze CAZ at much lower extent than ESBLs, thus generating very low MICs (1, 4). Nevertheless, in our previous analysis, we showed that CAZ cannot be used to make this distinction (3). As depicted in Table 1, this phenomenon may have two explanations. First, in our data set, only CTX-M-15 producers demonstrated high MICs for CAZ (MIC_90s_ of 32 µg/mL), whereas those expressing CTX-M-1 had MIC_90s_ of 4 µg/mL; this is also consistent with the findings from other authors (File S2) (7, 23). Second, *Ko*C strains hyperproducing WT OXYs showed CAZ MIC_90s_ of 2 µg/mL, but those producing the OXY variants have MICs similar to those of the CTX-M-15 producers (e.g., strain R1057, MIC of 128 µg/mL) (1, 5, 6).

Using the BMD method against our 44 *Ko*C strains, the mutual CAZ EUCAST/CLSI screening cutoff (MIC >1 µg/mL) assured 100% Se along with good Sp and PPV (84.6% and 64.7%, respectively) (Table 3). This cutoff, when hypothetically implemented, also assured the same performance with the *Ko*C strains previously described by others (File S1) (7, 23, 34, 41, 43). However, in both VITEK cards tested, the >1 µg/mL cutoff did not perform well (~65%–73% Se) (Table 3). Therefore, we advise no deviation from the present CAZ EUCAST/CLSI cutoff (Table 4), but we emphasize that adequate screening for potential ESBL producers might not be ideal using VITEK cards.

EUCAST suggests screening for ESBL producers when the inhibition zone for the 10 µg disk is ≤21 mm (28). For our *Ko*C strains, this approach showed 91.7% Se, 73.3% Sp, and 47.8% PPV. In contrast, a slightly higher cutoff of ≤22 mm could offer 100% Se, although the decrease of Sp and PPV may raise the number of ESBL-CTs of ~4%–5% (Table 3). With regard to the CLSI, the CAZ disk (30 µg) cutoff is set at ≤22 mm (19), resulting in a very low accuracy for our collection of strains (61.1% Se, 94.3% Sp). We note that only by using a ≤29 mm cutoff, a 100% Se could be reached, but with poor Sp and PPV (66.0% and 39.3%, respectively) (Table 3). This observation was also supported by Fujita et al., who reported CAZ (30 µg) inhibition diameters for the CTX-M producers of 24 and 26 mm, while those for non-ESBL-positive strains were >22 mm in ~60% of the cases (File S2) (17).

Overall, we propose screening cutoffs of ≤22 and ≤30 mm for the EUCAST and CLSI CAZ disks for *Ko*C, respectively. However, we underline that the use of the 30 µg CLSI disk with the ≤30 mm cutoff would imply testing ~70% more *Ko*C strains (Table 4). This lower performance of the CLSI disk was already noted for the ESBL confirmatory CDT executed in the present analysis (see above; Tables 1 and 2).

### Screening cutoffs using CTX

Although CTX is historically proposed by both EUCAST and CLSI to screen for suspicious ESBL producers (18, 19), the two VITEK cards implemented in the present study do not test for this substrate. Using the BMD method, the common EUCAST/CLSI cutoff (>1 µg/mL) for CTX assured 100% Se, but Sp and PPV were 73.3% and 47.8%, respectively. On the other hand, increasing the cutoff to >4 µg/mL could offer 100% Se, 94.3% Sp, and 84.6% PPV (~23% of unnecessary ESBL-CTs saved) for our collection of *Ko*C strains (Table 3). Of note, the great majority of the hOXY- and WT-*Ko*C strains described in previous studies demonstrated CTX MICs of ≤2 µg/mL (File S2) (23, 41). Therefore, we believe that a MIC of CTX >4 µg/mL represents an ideal cutoff that can be implemented to screen for suspicious ESBL-*Ko*C strains (Table 4).

When tested against our strains collection, the EUCAST CTX disk (5 µg) screening cutoff set at ≤20 mm resulted in 100% Se, but only 62.3% Sp and 35.5% PPV (28). On the other hand, a cutoff of ≤12 mm could offer 100% Se, 89.2% Sp, and 73.3% PPV. This lower cutoff could also decrease the number of ESBL-CTs to ~35% (Table 3). Concerning the 30 µg CTX disk, the CLSI recommends a ≤27 mm cutoff that performed relatively well with our tested strains (100% Se, 78.6% Sp, and 55% PPV). However, a ≤22 mm cutoff not only could assure 100% Se, but also excellent Sp and PPV (94.3% and 84.6%, respectively) (Table 3). In this context, Fujita et al. reported inhibition diameters of 15 and 18 mm for the two CTX-M producers, while >22 mm for 67% of the non-ESBL-positive strains (File S2) (17). Overall, when testing *Ko*C strains, we propose ESBL screening cutoffs of ≤12 and ≤22 mm for the EUCAST and CLSI CTX disks, respectively (Table 4).

### Screening cutoffs using ATM

ATM is not included in both VITEK cards utilized in the present work. By using the BMD method (File S1), the CLSI cutoff (MIC >1 µg/mL) resulted in 100% Se, but low Sp and PPV (58.9% and 32.4%, respectively); alternative cutoffs of >2 and >4 µg/mL could also not improve the screening performance for our *Ko*C strains (Table 3).

With regard to the disk diffusion, the CLSI cutoff of ≤27 mm demonstrated 91.7% Se and 60% Sp. On the other hand, a slightly higher cutoff of ≤28 mm could assure 100% Se, but still offering very low Sp and PPV (60% and 33%, respectively). Notably, in the study of Fujita et al., all hOXY-*Ko*C strains showed inhibition zone diameters for ATM of ≤17 mm (File S2) (17).

Overall, we suggest that the MIC screening cutoff for ATM remain at >1 µg/mL (as suggested by CLSI), whereas that for the 30 µg disk should be increased to ≤28 mm (Table 4). Importantly, since both methods offer very low Sp and PPV, we do not recommend using ATM for the ESBL screening of *Ko*C strains.

### Conclusions

The VITEK 2 equipped with the AES performed poorly, whereas VITEK 2 alone followed by the CLSI ESBL screen and confirmatory tests yielded a reliable methodology to distinguish between ESBL-*Ko*C and hOXY-*Ko*C. In this context, we suggest the use of the CTX/CTX-CL and CAZ/CAZ-CL CDT ESBL-CTs, with no preferences between CLSI- or EUCAST-recommended and differently loaded disks as long as there are no specific studies for *Ko*C strains demonstrating any possible difference in their analytic performance.

Nevertheless, this overall strategy also presents two drawbacks: it increases the turnaround time for the final report and implies a high number of unnecessary ESBL-CTs, thus affecting the workload for the laboratory technicians and overall diagnostic costs. In order to solve the latter issue, this study also revised the screening cutoffs for CRO, CPD, CAZ, CTX, and ATM to negate unnecessary ESBL-CTs for *Ko*C strains. These cutoffs assured 100% Se and higher Sp/PPV compared to the current CLSI/EUCAST screening criteria when implemented with our *Ko*C collection. However, the proposed cutoffs were obtained from testing only 44 strains, which, although well-defined, may lack the statistical power to ensure optimal results. Therefore, our findings should be validated and verified using larger and well-characterized *KoC* collections producing a broader range of ESBL and OXY β-lactamases.

## SUPPLEMENTAL MATERIAL

File S1 jcm.00128-25-s0001.xlsx Epidemiology, phenotypes, WGS, confirmatory tests, and Vitek cards.

File S2 jcm.00128-25-s0002.xlsx Phenotypic and confirmatory tests from literature.

ASM does not own the copyrights to Supplemental Material that may be linked to, or accessed through, an article. The authors have granted ASM a non-exclusive, world-wide license to publish the Supplemental Material files. Please contact the corresponding author directly for reuse.
